# Exploration of NO_2_ and PM_2.5_ air pollution and mental health problems using high-resolution data in London-based children from a UK longitudinal cohort study

**DOI:** 10.1016/j.psychres.2018.12.050

**Published:** 2019-02

**Authors:** Susanna Roberts, Louise Arseneault, Benjamin Barratt, Sean Beevers, Andrea Danese, Candice L. Odgers, Terrie E. Moffitt, Aaron Reuben, Frank J. Kelly, Helen L. Fisher

**Affiliations:** a King's College London, Social, Genetic & Developmental Psychiatry Centre, Institute of Psychiatry, Psychology & Neuroscience, 16 De Crespigny Park, SE5 8AF, London, UK; b King's College London, Environmental Research Group, MRC-PHE Centre for Environment and Health, London, UK; c King's College London, Department of Child & Adolescent Psychiatry, Institute of Psychiatry, Psychology & Neuroscience, London, UK; d National & Specialist CAMHS Clinic for Trauma, Anxiety and Depression, South London & Maudsley NHS Foundation Trust, London, UK; e Sanford School of Public Policy, Duke University, Durham, NC, USA; f Department of Psychology and Social Behavior, University of California Irvine, Irvine, CA, USA; g Departments of Psychology and Neuroscience, Psychiatry and Behavioral Sciences, and Centre for Genomic and Computational Biology, Duke University, Durham, NC, USA

**Keywords:** ADHD, Anxiety, Conduct disorder, Depression, Environment, Neighbourhood, Psychiatric, Psychopathology

## Abstract

Air pollution is a worldwide environmental health issue. Increasingly, reports suggest that poor air quality may be associated with mental health problems, but these studies often use global measures and rarely focus on early development when psychopathology commonly emerges. To address this, we combined high-resolution air pollution exposure estimates and prospectively-collected phenotypic data to explore concurrent and longitudinal associations between air pollutants of major concern in urban areas and mental health problems in childhood and adolescence. Exploratory analyses were conducted on 284 London-based children from the Environmental Risk (E-Risk) Longitudinal Twin Study. Exposure to annualized PM_2.5_ and NO_2_ concentrations was estimated at address-level when children were aged 12. Symptoms of anxiety, depression, conduct disorder, and attention-deficit hyperactivity disorder were assessed at ages 12 and 18. Psychiatric diagnoses were ascertained from interviews with the participants at age 18. We found no associations between age-12 pollution exposure and concurrent mental health problems. However, age-12 pollution estimates were significantly associated with increased odds of major depressive disorder at age 18, even after controlling for common risk factors. This study demonstrates the potential utility of incorporating high-resolution pollution estimates into large epidemiological cohorts to robustly investigate associations between air pollution and youth mental health.

## Highlights

• High-resolution pollution estimates were successfully combined with cohort data.
• Age-12 pollution exposure was not associated with age-12 mental health problems.
• But age-12 pollution exposure was significantly associated with age-18 depression.
• Associations with depression held even after controlling for common risk factors.
• Elevated odds of age-18 conduct disorder among children exposed to air pollution.

## 1 Introduction

Mental health problems are diagnosed in 10–20% of children and adolescents worldwide (Polanczyk et al., 2015). Incidence and typical age of onset varies by diagnosis, but all can have negative impacts on numerous facets of life including daily functioning, social interactions, and educational achievement (e.g. McLeod and Kaiser, 2004, Parker et al., 2015, Van Ameringen et al., 2003). Research has demonstrated that the majority of adult psychopathologies begin in childhood and adolescence (Kim-Cohen et al., 2003), with the poorest prognosis for those whose problems begin early and persist (Copeland et al., 2015, Kessler et al., 2007). The identification of early risk factors may inform the development of interventions to prevent the emergence of mental health problems in the first two decades of life.

Numerous factors involved in the etiology of child and adolescent mental health have been identified and well-validated, including family psychiatric history (e.g. Rasic et al., 2013, Silberg et al., 2012) and exposure to victimization in childhood (e.g. Arseneault et al., 2011, Jaffee et al., 2004, Norman et al., 2012). However, the role of the wider environment is less clear. Previous reports have demonstrated that cities have an elevated prevalence of adult psychiatric diagnoses (Peen et al., 2010, Sundquist et al., 2004, Vassos et al., 2016) and child and adolescent mental health problems (Newbury et al., 2018, Newbury et al., 2016, Rudolph et al., 2014), with a number of environmental factors suggested to account for the differences between urban and rural environments, including poor air quality. Air pollution is a worldwide environmental health issue (Health Effects Institute 2010, World Health Organisation 2013), but is a particular concern in urban environments, especially large cities such as London (UK) where air quality is substantially lower. Despite some improvement in recent years due to increased regulation of vehicle emissions, air pollution levels in London are consistently higher than the limits set by the European Union (EU) and WHO guidelines (Beevers et al., 2016). Furthermore, research suggests that the risk of adverse outcomes is significantly elevated even at levels below legal limits (Beelen et al., 2013), suggesting that there may be no “safe” level for air pollution exposure (WHO, 2013). Substantial variability in air pollution concentrations exists in large cities, and comprehensive data for London is available via the KCLurban air pollution exposure model at a resolution of 20 m × 20 m and based on measured air pollution from several sources (Kelly et al., 2011). Thus, London is an ideal city in which to explore associations between air pollution exposure and the development of mental health problems.

Research has demonstrated that exposure to high levels of air pollution is a strong risk factor for poor cardiovascular and respiratory outcomes (Kelly and Fussell, 2015, Rückerl et al., 2011), partly resulting from inflammation and oxidative stress in exposed organ systems (Kelly, 2003, Laumbach et al., 2014). Inflammatory processes have been suggested to play a crucial role in the etiology of a wide range of psychiatric diagnoses (Coccaro et al., 2014, Miller and Raison, 2016, Najjar et al., 2013), suggesting a link between air pollution exposure and mental health problems is plausible (Guxens and Sunyer Deu, 2012). Experimental research in animals (Levesque et al., 2011) and post-mortem observations in humans (Calderón-Garcidueñas et al., 2008) have demonstrated that air pollutants, particularly fine and ultrafine particles, are capable of reaching the brain, potentially by crossing the blood-brain-barrier or translocation along the olfactory nerve. Once in the brain, pollutant particles can modulate vasoregulatory pathways and trigger neuroinflammation (Block and Calderón-Garcidueñas, 2009). Indeed, exposure to inflammatory stimuli may have an even greater impact in childhood and adolescence owing to ongoing brain development (Danese and Baldwin, 2017). A growing number of reports have demonstrated associations between air pollution and psychopathology. A large study in adults found an association between exposure to PM_2.5_ (particulate matter less than 2.5 µm in diameter) and anxiety symptom scores (Power et al., 2015), while associations have also been reported for depression (Lim et al., 2012, Szyszkowicz et al., 2009), and suicidality (Bakian et al., 2015, Kim et al., 2010). However, one of the largest studies to date that explored associations between air pollution exposure and depression in adult general population samples from 4 different European countries reported inconsistent results between samples (Zijlema et al., 2016).

Children may be especially susceptible to neurologic injury from air pollution because their brains are still developing (Rice and Barone, 2000), and they are likely to have less established natural barriers in the lungs to protect against inhaled particles (Brockmeyer and D'Angiulli, 2016). In addition, children have a higher breathing rate to body size ratio, and tend to spend more time outdoors than adults, further increasing their risk of exposure to air pollution (Brockmeyer and D'Angiulli, 2016). A handful of existing studies have reported associations between air pollution exposure and higher rates of attention-deficit hyperactivity disorder (ADHD; Min and Min, 2017, Newman et al., 2013, Siddique et al., 2010), autism (Becerra et al., 2013), anxious/depressive symptoms (Perera et al., 2012), and behavioral problems (Forns et al., 2016; Yorifuji et al., 2017) in children. A recent population-based study reported increased rates of dispensed psychiatric medication amongst children and adolescents living in areas with higher air pollution concentrations (Oudin et al., 2016). Whilst indirect measures such as medication use can powerfully maximize sample size in population studies, it is a crude measure which includes a wide range of dispensed medications, for a large spectrum of mental health problems of varying severities, and is not consistent across consecutive years. These factors may limit the conclusions that can be drawn from such studies, as mental health problems may be under-detected, causing a bias in the results, and the association with specific mental health outcomes cannot be inferred. In order to extend these findings, it is crucial to use more comprehensive and direct measures of psychopathology as well as higher resolution estimates of pollution exposure. Though some previous studies have used high-quality personal monitoring of air pollutant exposures (e.g. Perera et al., 2013), most existing studies have tended to rely on relatively low resolution, regional-level pollution exposure data. Given the potential for variability in short-range pollutant levels within urban centres, such as London, these measures may not be sufficient to accurately capture personal levels of exposure. Additionally, longitudinal samples are required to better tease out the temporal relationship between pollution exposure and development of mental health problems, and assess the potential longer-term effects of early and/or cumulative exposure to pollutants. Indeed, results in adult samples suggest that longer durations of exposure to pollutants are associated with more adverse outcomes (e.g. Brunekreef et al., 2009).

The aim of this exploratory study was to investigate potential associations between estimated exposure to ambient air pollution in late childhood and mental health problems assessed concurrently in childhood and prospectively in late adolescence. To date, associations between exposure to air pollution and a range of mental health outcomes have not been explored in a longitudinal UK-based sample of children and adolescents. We utilized previously modelled high-resolution annualized average concentration estimates based on air pollution measurements from several sources for a major urban center (London, UK), focusing on two air pollutants (PM_2.5_ and NO_2_). We undertook a novel integration with a well-established UK population-based birth cohort study, which comprised previously collected comprehensive assessments of major mental health problems at ages 12 and 18. We then explored whether these air pollutants were associated with concurrent and longitudinal mental health problems, and checked the robustness of associations for other important risk factors, including socioeconomic status, family psychiatric history, childhood victimization, and smoking, as well as controlling for age-12 symptoms in age-18 analyses. We hypothesized that estimated exposure to higher levels of PM_2.5_ and NO_2_ would be associated with elevated rates of mental health problems concurrently at age 12 and longitudinally in late adolescence (age 18), and that these associations would hold after adjustment for key confounders.

## 2 Methods

### 2.1 Sample

The sample for this analysis was drawn from the Environmental Risk (E-Risk) Longitudinal Twin study, a nationally-representative sample of twins born in 1994 and 1995 in England and Wales (described in full elsewhere; Moffitt and the E-Risk Study Team, 2002). Briefly, the E-Risk sample was constructed in 1999–2000, when 1116 families with same-sex 5-year-old twins (93% of those eligible) participated in home-visit assessments. The full sample comprised 56% monozygotic (MZ) and 44% dizygotic (DZ) twin pairs; sex was evenly distributed within zygosity (49% male). Families were recruited to represent the UK population of families with newborns in the 1990s, based on residential location throughout England and Wales and mothers’ age (teenaged mothers with twins were over-selected to replace high-risk families who were selectively lost to the register through non-response. Older mothers having twins via assisted reproduction were under-selected to avoid an excess of well-educated older mothers). The study sample represents the full range of socioeconomic conditions in Great Britain, as reflected in the families’ distribution on a neighborhood-level socioeconomic index (ACORN [A Classification of Residential Neighborhoods], developed by CACI Inc. for commercial use) (Odgers et al., 2012). E-Risk families’ ACORN distribution closely matches that of households nation-wide: 25.6% of E-Risk families live in “wealthy achiever” neighborhoods compared to 25.3% nationwide; 5.3% vs. 11.6% live in “urban prosperity” neighborhoods; 29.6% vs. 26.9% live in “comfortably off” neighborhoods; 13.4% vs. 13.9% live in “moderate means” neighborhoods; and 26.1% vs. 20.7% live in “hard-pressed” neighborhoods. E-Risk underrepresents “urban prosperity” neighborhoods because such households are likely to be childless.

Follow-up home visits were conducted when the participants were aged 7 (98% participation), 10 (96%), 12 (96%), and at 18 years (93%). Home visits at ages 5, 7, 10, and 12 years included assessments with participants as well as their primary caretaker; the home visit at age 18 included interviews only with the participants. Each twin participant was assessed by a different interviewer. With the parent's permission, questionnaires were mailed to the children's teachers when they were 7 years (93% response rate), 10 years (90%) and 12 years (80%). The Joint South London and Maudsley and the Institute of Psychiatry Research Ethics Committee approved each phase of the study. Parents gave informed consent and twins gave assent between 5–12 years and informed consent at age 18.

The subsample utilized in this analysis comprised 284 children living in London in 2007. On average, the study participants were aged 12, and 54% of the sample was male. There were no significant differences in gender, zygosity, or victimization exposure between this London-based subsample and the rest of the E-Risk sample, but differences were observed for ethnicity, family-level socioeconomic status (SES), neighborhood-level SES, smoking, and family psychiatric history (Supplementary Table 1). Unsurprisingly, in this London subsample, the ‘urban prosperity’ category of neighborhood SES was over-represented (23.4% vs. 11.6% nation-wide), whereas it was under-represented in the full E-Risk sample (5.3%).

**Table 1:** The association between air pollution estimates for 2007 (age 12) and continuous phenotypic outcomes at age 12.

Pollutant	Model	Anxiety			Depression			Conduct disorder			ADHD		
		**β**	**95% CI**	***p***	**β**	**95% CI**	***p***	**β**	**95% CI**	***p***	**β**	**95% CI**	***p***
**PM_2.5_**	Basic^a^	0.01	−0.13–0.15	0.927	0.06	−0.07–0.19	0.373	0.11	−0.08–0.31	0.246	0.12	−0.04–0.27	0.155
	Full^b^	−0.04	−0.19–0.11	0.582	0.00	−0.15–0.14	0.951	0.09	−0.10–0.28	0.355	0.05	−0.11–0.20	0.542
**NO_2_**	Basic^a^	−0.01	−0.14–0.13	0.923	0.06	−0.06–0.18	0.327	0.11	−0.07–0.29	0.212	0.11	−0.04–0.26	0.159
	Full^b^	−0.06	−0.20–0.09	0.454	0.00	−0.14–0.14	0.999	0.09	−0.09–0.27	0.308	0.04	−0.10–0.19	0.561

ADHD, attention deficit hyperactivity disorder. β, standardized beta coefficient from linear regression; β coefficients depict the unit SD change in phenotype given 1 SD change in air pollution estimates. CI, confidence interval.

a Models were adjusted for the confounding effects of sex, ethnicity, and neighborhood socioeconomic status (SES), as well as the non-independence of twin observations.

b Models were adjusted for the confounding effects of sex, ethnicity, neighborhood SES, family SES, family psychiatric history, and exposure to severe childhood victimization, as well as the non-independence of twin observations.

### 2.2 Measures

#### 2.2.1 Air pollution estimates

Pollution exposure estimates were based on the latitude-longitude coordinates of the twins’ residential addresses in 2007, when the twins were aged 12 years, and derived at a 20 m × 20 m resolution from the KCLurban model. This model is well-established for use in public health research (Kelly et al., 2011) and performed well in the UK Model Inter-Comparison Exercise, run by King's on behalf of DEFRA (http://uk-air.defra.gov.uk/library/reports?report_id=777). Full details of the modelling methodology and evaluation of its performance are provided in Kelly et al. (2011). Briefly, the KCLurban model used a kernel modelling technique, based upon the ADMS (CERC, 2006), to describe the initial dispersion from each emissions source. The contribution from each source was summed onto a fixed 20 m × 20 m grid across London assuming that one can calculate the contribution of any source to total air pollution concentrations by applying each kernel and adjusting for the source strength. The kernels were produced using an emissions source of unity, either 1 gs^−1^ (point and jet sources), 1 gm^−3^s^−1^ (volume sources) or 1 gkm^−1^s^−1^ (road sources) and hourly meteorological measurements from the UK Meteorological Office site at Heathrow. Data from the Heathrow site were recorded at a height of 10 m and included measurements of temperature, wind speed, wind direction, precipitation, relative humidity and cloud cover. The KCLurban model used emissions from the London Atmospheric Emissions Inventory (LAEI) and for road traffic emissions using King's well-established emissions modelling methods, combined with emissions factors from the UK specific roadside measurements of Carslaw et al. (2011) and for non-exhaust emissions, based upon the work of Harrison et al. (2012). Concentration estimates were obtained for 8 pollutants (NO, NO_2_, NO_x_, O_3_, O_x_, PM_10_, PM_2.5_, and PMcoarse [PM_10_–PM_2.5_]). High correlations were seen between all air pollution concentration estimates (all r's > 0.9), and as such analyses in this paper focus on two pollutants to minimize multiple testing: PM_2.5_ and NO_2,_ which are known to cause adverse health effects and have been previously evaluated for association with mental health outcomes (Costa et al., 2014).

#### 2.2.2 Age-12 mental health symptoms

Depression and anxiety symptoms were assessed using the Children's Depression Inventory (CDI; Kovacs, 2004) and the Multidimensional Anxiety Scale for Children (MASC; March, 1997) respectively, in private interviews. Conduct disorder symptoms were based on self-report according to DSM-IV criteria (American Psychiatric Association, 1994). ADHD symptoms at age 12 were ascertained via by mothers’ and teachers’ reports of inattention and hyperactivity-impulsivity according to DSM-IV criteria and the Rutter Child Scales (American Psychiatric Assocation 1994, Kelly et al., 2011). Descriptive statistics are provided in Supplementary Table 2.

**Table 2:** The association between air pollution estimates for 2007 (age 12) and continuous phenotypic outcomes at age 18.

Pollutant	Model	Anxiety			Depression			Conduct disorder			ADHD		
		**β**	**95% CI**	***p***	**β**	**95% CI**	***p***	**β**	**95% CI**	***p***	**β**	**95% CI**	***p***
**PM_2.5_**	Basic^a^	0.02	−0.16–0.20	0.822	**0.20**	**0.03–0.34**	**0.019**	0.10	−0.07–0.27	0.244	0.08	−0.12–0.28	0.424
	Full^b^	−0.01	−0.19–0.18	0.917	0.16	−0.00–0.33	0.056	0.09	−0.08–0.26	0.294	0.04	−0.14–0.21	0.685
**NO_2_**	Basic^a^	0.02	−0.15–0.19	0.812	**0.20**	**0.04–0.37**	**0.017**	0.11	−0.05–0.28	0.186	0.08	−0.11–0.28	0.411
	Full^b^	−0.01	−0.18–0.16	0.902	0.17	0.00–0.34	0.050	0.10	−0.06–0.27	0.221	0.04	−0.13–0.21	0.644

ADHD, attention deficit hyperactivity disorder. β, standardized beta coefficient from linear regression; β coefficients depict the unit SD change in phenotype given 1 SD change in air pollution estimates. CI, confidence interval. Significant results at *p *< 0.05 are in bold.

a Models were adjusted for the confounding effects of sex, ethnicity, neighborhood socioeconomic status (SES), and ever a daily smoker, as well as the non-independence of twin observations.

b Models were adjusted for the confounding effects of sex, ethnicity, neighborhood SES, family SES, family psychiatric history, exposure to severe childhood victimization, relevant age-12 mental health problem (i.e. anxiety, depression, conduct disorder, or ADHD), and ever a daily smoker, as well as the non-independence of twin observations.

#### 2.2.3 Age-18 mental health diagnoses

Participants reported on symptoms of major depressive disorder, generalized anxiety disorder, and conduct disorder at age 18. The exception was ADHD symptoms, which for consistency with the measure of ADHD symptoms at age 12, were taken from a composite measure of reported symptoms by two co-informants (usually the mother and co-twin; more information available in Agnew-Blais et al., 2016). Diagnoses were made according to DSM-IV and DSM-V (ADHD) criteria following private interviews with trained researchers using the Diagnostic Interview Schedule (Robins et al., 1995). Supplementary Table 2 provides the descriptive statistics, which show a reasonable degree of variability. At age 18, 6.4% of the subsample met DSM-IV criteria for anxiety disorder, 18.8% for major depressive disorder, 15.6% for conduct disorder, and 6.8% for ADHD (DSM-V criteria). These rates are comparable to those for the full E-Risk sample which is spread across England and Wales: anxiety disorder −7.4%, major depressive disorder −20.1%, conduct disorder −15.0%, and ADHD −8.3%.

#### 2.2.4 Confounders

The effects of sex, ethnicity, neighborhood SES, family SES, family psychiatric history, exposure to severe victimization, and smoking were considered as confounders due to their potential associations with pollution exposure and/or phenotypic outcomes. Family SES was determined using a standardized composite variable including total household income, parents’ highest education level, and parents’ highest occupational grade when children were aged 5. These three SES indicators were highly correlated (*r *= 0.57–0.67, all *p*’s < 0.05) and loaded significantly onto one latent factor (factor loadings = 0.80, 0.70, and 0.83 for income, education, and social class, respectively) (Trzesniewski et al., 2006). The population-wide distribution of this latent factor was then divided into tertiles to facilitate meaningful comparisons (i.e. low-, medium-, and high-SES). In this subsample, 26.8% were classified as low SES. Neighborhood SES was classified using ACORN for the E-Risk families’ postcodes when children were aged 12 (Odgers et al., 2012) as described earlier. Mothers reported family history of DSM diagnoses in private interviews (Weissman et al., 2000), which was converted to a proportion (0–1.0) of family members with a history of psychiatric diagnoses (Milne et al., 2008). Victimization during childhood (25.7%) was classified as severe exposure up to 12 years of age to any of 6 domains of victimization (domestic violence between the mother and her partner; frequent bullying by peers; physical maltreatment by an adult; sexual abuse; emotional abuse or neglect; and physical neglect), based on caregiver reports (usually the mother), recorded narratives of the caregiver interviews, home observations by the research workers, child self-reports (bullying), and information from clinicians whenever the study team made a child-protection referral (see Danese et al., 2016 and Fisher et al., 2015). Smoking status was determined based on whether the participant reported ever having been a daily smoker (yes/no) when questioned at age 18; 20% of this subsample met this criterion.

### 2.3 Statistical analyses

All analyses were conducted in Stata v14.0 (Stata Corp., College Station, Tex.). Linear regression was used to investigate the association between estimated levels of the two pollution concentrations and continuous symptom outcomes. Binary logistic regression was used to test the association between pollution estimates and diagnostic outcomes. Pollution estimates and continuous measures of mental health symptoms were standardized to allow for comparison of model coefficients. All analyses were adjusted to account for the non-independence of twin observations using the Huber–White variance estimator (Williams, 2000). Application of this technique allows for the relaxation of the assumption of independence of observations by penalizing estimated standard errors and therefore accounting for the dependence in the data due to analysing sets of twins.

Firstly, we used cross-sectional analysis to examine the associations between pollution estimates in 2007 (when children were aged 12) and concurrent symptoms of mental health problems in the same year. Secondly, we tested prospective associations between pollution exposure in 2007 (at age 12), with continuous symptoms and diagnoses at age 18. Thirdly, we repeated the analyses including family-level covariates (family SES and family psychiatric history), childhood victimization exposure, and for age-18 models we further controlled for daily smoking and the relevant age-12 mental health problem (i.e. age-12 depression when investigating associations with age-18 depression). In all analyses, the mental health measure of interest was the dependent variable, the (standardized) pollutant was the independent variable, and sex, ethnicity (coded as white and non-white), and neighborhood SES were included as covariates. Tests were interpreted as significant at *p *< 0.05 due to the exploratory nature of this study.

## 3 Results

### 3.1 Air pollution exposure

**Fig. 1:**
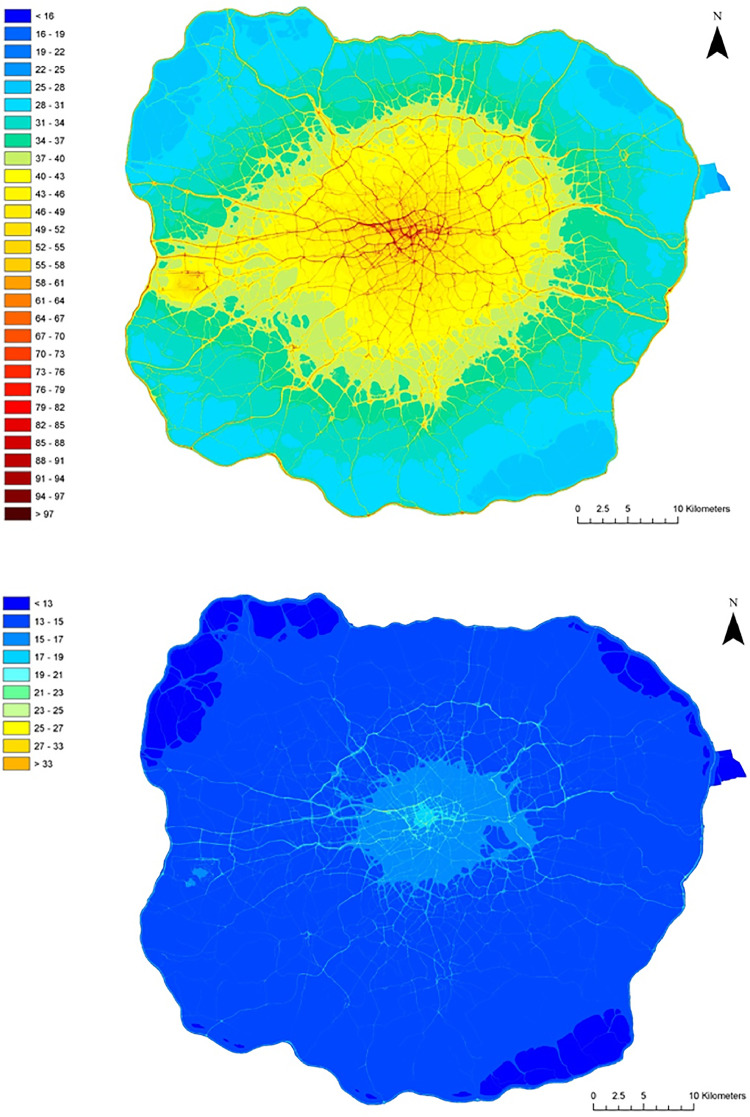

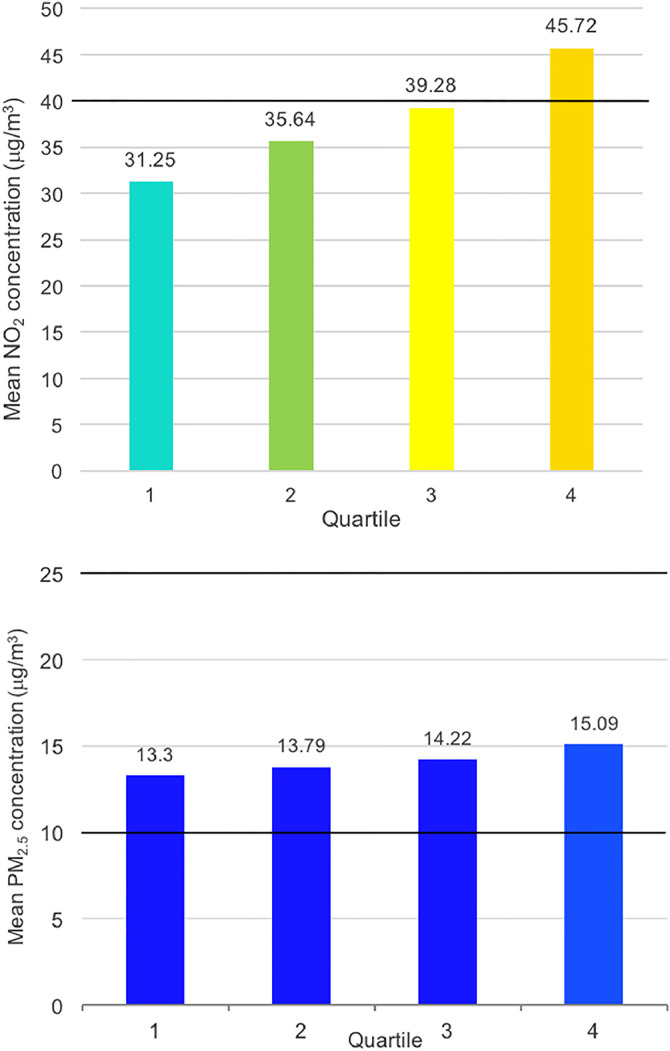
(a) Annual mean NO2 and PM2.5 for London in 2007. Figures display the annual mean NO_2_ (left) and PM_2.5_ (right) concentrations in 2007 across London. Color scale indicates compliance with national standards, with yellow and above indicating non-compliance. (b) Annual mean NO_2_ and PM_2.5_ for the E-Risk sample in 2007. Graphs display the annual mean NO_2_ (left) and PM_2.5_ (right) concentration in 2007 by quartile (μg/m^3^) for the parts of London where the E-Risk participants were living during this year. The black lines denote the current EU legislated standard and WHO air quality guidelines (40 μg/m^3^ for NO_2_; 25 μg/m^3^ and 10 μg/m^3^ for PM2.5 respectively).

The annualized average concentration estimate for PM_2.5_ was 14.09 μg/m^3^ (SD = 0.69, IQR 13.6–14.52). The annualized average concentration estimate for NO_2_ was 37.9 μg/m^3^ (SD = 5.5, IQR 34.1–41.7). In this sample, annualized pollution estimates for NO_2_ were above the current EU legislated standard for 31% of children (40 μg/m^3^; also the current WHO air quality guideline). For PM_2.5_, annualized estimates were within the current specified EU range for PM_2.5_ (25 μg/m^3^), but exceeded the WHO air quality guideline for all children (10 μg/m^3^). Fig. 1a shows the distribution of annualized estimates for PM_2.5_ and NO_2_ in 2007 in London, and Fig. 1b the average mean concentration in quartiles based on the participants’ home addresses in this year. Over two-thirds of the subsample (66.7%) did not move house in the preceding years (ages 5 to 12), indicating exposure to similar levels of air pollution throughout childhood is likely. Exposure to air pollution varied significantly by neighborhood level SES for both pollutants (both *p*’s < 0.001). Those in neighborhoods classified as ‘urban prosperity’ and ‘hard-pressed’ were exposed to the highest average annualized pollution levels, whilst those in neighborhoods classified as ‘wealthy achievers’ had the lowest average annualized pollution estimates (Supplementary Table 3).

**Table 3:** The association between air pollution estimates for 2007 (age 12) and psychiatric diagnoses at age 18.

Pollutant	Model	Anxiety			Depression			Conduct disorder			ADHD		
		**OR**	**95% CI**	***p***	**OR**	**95% CI**	***p***	**OR**	**95% CI**	***p***	**OR**	**95% CI**	***p***
**PM_2.5_**	Basic^a^	1.14	0.52–2.52	0.737	**1.69**	**1.13–2.53**	**0.010**	1.74	0.97–3.13	0.062	1.52	0.93–2.49	0.093
	Full^b^	1.05	0.45–2.44	0.916	**1.63**	**1.08–2.46**	**0.021**	1.73	0.95–3.15	0.074	1.16	0.64–2.10	0.619
**NO_2_**	Basic^a^	1.13	0.51–2.49	0.771	**1.64**	**1.11–2.42**	**0.014**	1.76	1.00–3.11	0.052	1.50	0.93–2.42	0.094
	Full^b^	1.02	0.44–2.35	0.969	**1.57**	**1.05–2.35**	**0.029**	1.75	1.00–3.07	0.050	1.20	0.69–2.09	0.526

ADHD, attention deficit hyperactivity disorder. OR, Odds Ratio from logistic regression. CI, confidence interval. Significant results at *p *< 0.05 are in bold.

a Models were adjusted for the confounding effects of sex, ethnicity, neighborhood socioeconomic status (SES), and ever a daily smoker, as well as the non-independence of twin observations.

b Models were adjusted for the confounding effects of sex, ethnicity, neighborhood SES, family SES, family psychiatric history, exposure to severe childhood victimization, relevant age-12 mental health problem (i.e. anxiety, depression, conduct disorder, or ADHD), and ever a daily smoker, as well as the non-independence of twin observations.

### 3.2 Concurrent associations between exposure to pollution and symptoms of mental health problems

We found no significant associations between pollution estimates and concurrent symptoms of mental health problems at age 12 and all effect sizes were small (Table 1).

### 3.3 Longitudinal associations between exposure to pollution and diagnoses of mental health problems in young adulthood

Associations between both PM_2.5_ and NO_2_ exposure at age 12 and symptoms of depression at age 18 were statistically significant in the basic model, but were attenuated by the inclusion of further covariates in the full model (Table 2). Effect sizes suggested potential associations between PM_2.5_ and NO_2_ exposure and symptoms of conduct disorder, but these were not statistically significant (*p > *0.05, Table 2). No significant associations were detected between pollution estimates and later symptoms of anxiety or ADHD (Table 2).

**Fig. 2:**
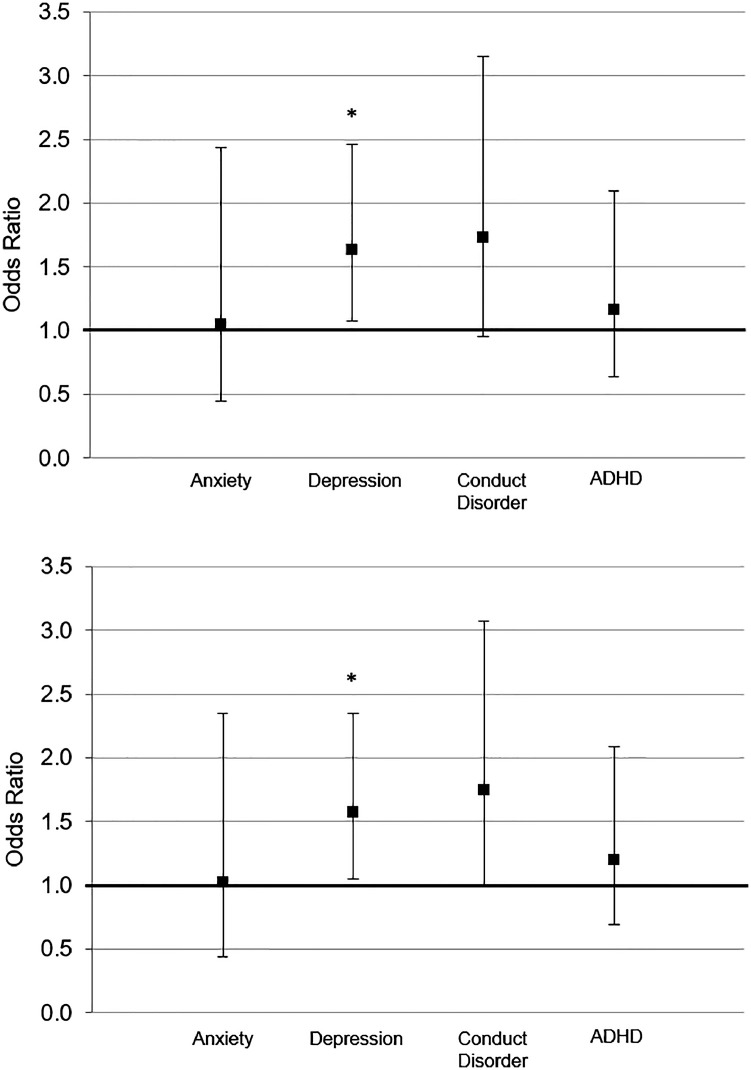
The association between air pollution estimates (a) PM_2.5_ and (b) NO_2_ for 2007 (age 12) and psychiatric diagnoses at age 18. Graphs depict odds ratios with 95% confidence intervals. Models were adjusted for the confounding effects of sex, smoking, neighborhood socioeconomic status (SES), family SES, family psychiatric history, childhood victimization, and the relevant age-12 mental health problem (i.e. anxiety, depression, conduct disorder, or ADHD), as well as the non-independence of twin observations. ADHD, attention deficit hyperactivity disorder. * *p *< 0.05.

Annualized estimates for PM_2.5_ and NO_2_ concentrations were both significantly associated with a diagnosis of major depressive disorder at age 18 in the basic model (Table 3), and even when controlling for neighborhood SES, family-level covariates, childhood victimization, smoking, and age-12 depressive symptoms (Fig. 2a andb). Annualized estimates for PM_2.5_ and NO_2_ concentrations were both associated with increased odds of a diagnosis of conduct disorder at age 18 (Table 3), even after adjustment for confounders (Fig. 2a and b), but these associations failed to reach conventional levels of statistical significance. Odds ratios for both pollutants were greater than 1, indicating increased odds of depression and conduct disorder at age 18 when exposed to higher estimated NO_2_ and PM_2.5_ levels at age 12. No associations were found between estimated exposure to PM_2.5_ or NO_2_ at age 12 and a diagnosis of anxiety disorder or ADHD at age 18 (all *p*’s > 0.05, Table 3).

### 3.4 Extremes analysis

When utilizing an extremes design, participants living in areas with the highest quartile of estimated pollution concentration showed a substantially elevated likelihood of depression at age 18 (after controlling for all previously described covariates) compared to those in the areas with the lowest estimated pollution levels (PM_2.5_: OR = 3.69, 95% CI 1.11–12.25, *p *= 0.033; NO_2_: OR = 4.08, 95% CI 0.90–18.60, *p *= 0.069). Elevated, though not significant, odds were also observed for conduct disorder (PM_2.5_: OR = 3.95, 95% CI 0.72–21.76, *p *= 0.114; NO_2_: OR = 5.12, 95% CI 0.68–38.58, *p *= 0.113). No associations were evident for anxiety or ADHD (all *p*’s > 0.4).

## 4 Discussion

This exploratory study demonstrates the feasibility of combining well-validated methods from multiple fields of research. Previously modelled high-resolution pollution estimates were linked with dense phenotyping data in an urban subsample of an established longitudinal epidemiological cohort study. Using this data, we investigated the associations between estimated air pollution exposure during childhood and both concurrent and later mental health problems. Cross-sectional analyses found no detectable associations between estimated pollution exposure and mental health problems at age 12, nor with anxiety or ADHD at age 18. However, estimated pollution concentration levels at age 12 were associated with an increase in symptoms and greater likelihood of a diagnosis of depression and conduct disorder at age 18, albeit the latter did not reach conventional levels of statistical significance. Elevated odds for depression and conduct disorder at age 18 were observed even when controlling for common risk factors including sex, ethnicity, smoking, neighborhood and family SES, family psychiatric history, victimization in childhood, and age-12 mental health problems. Of note, air pollution estimates were also associated with features of the local neighborhood; annualized mean concentrations showed a gradient of pollution exposure across neighborhood SES.

We found evidence for an association between exposure to higher levels of air pollution at age 12 and greater odds of developing depression at age 18. In the current study, individuals with the highest quartile of air pollution exposure were around 3–4 times more likely than those exposed to the lowest levels of pollution to be diagnosed with depression by the age of 18. To date, only one study has examined the relationship between any pollutant and depression in childhood or adolescence, finding that high prenatal exposure to prenatal polycyclic aromatic hydrocarbon (PAH) was associated with anxious/depressive symptoms at age 6–7 (Perera et al., 2012). Previous research in adult populations has produced inconsistent results in this area. Whilst depressive symptoms were found to be associated with short-term PM_10_, NO_2_ and O_3_ exposure in an elderly Korean sample (n = 537, Lim et al., 2012), and with PM_2.5_ exposure in a sample of older men (n = 4008, Pun et al., 2017), no effect of short-term ambient air pollution or long-term exposure to traffic pollution (proximity to major roads) was found in a sample of older adults in the Boston-area (n = 732, Wang et al., 2014). Short-term increases in air pollution have been associated with increases in emergency department visits for depression (Cho et al., 2014; Szyszkowicz et al., 2009), and perceived stress (Mehta et al., 2015). However, a recent large study of four European cohorts found inconsistent results for the effect of air pollution on depression (Zijlema et al., 2016), although one potential reason for this may be the use of different phenotypic definitions across the cohorts included (symptoms vs. diagnosis). Further research is thus required to examine different phenotypic expressions of mental health outcomes in the same individuals at different points in development to resolve these inconsistent findings.

We also found tentative evidence for an association between exposure to higher levels of air pollution at age 12 and greater odds of conduct disorder at age 18. Individuals with the highest quartile of air pollution exposure were around 3–5 times more likely than those exposed to the lowest levels of pollution to be diagnosed with conduct disorder by the age of 18. A small number of previous studies have examined the association between exposure to air pollution and externalizing symptoms such as conduct disorder. For instance, in a sample of school children in Barcelona, exposure to higher levels of traffic-related air pollutants was associated with increased behavioral difficulties but, as in the current study, not with ADHD symptomatology (Forns et al., 2016). Similarly, research from a Japanese nationally-representative longitudinal cohort reported an association between prenatal exposure to air pollution (PM_7_, NO_2_, and SO_2_) and behavioral difficulties at age 8 (Yorifuji et al., 2017). Previous research has also found that higher prenatal PAH exposure corresponded with reductions in white surface matter of (predominantly) the left hemisphere of the brain in childhood (Peterson et al., 2015). These structural differences were significantly associated with higher externalizing symptom scores, suggesting a potential mechanism by which exposure to certain pollutants may have an effect on behavioral problems in childhood.

Due to methodological differences between the current study and previous research, direct comparison of the observed effects is difficult. However, effect sizes for other environmental influences on both depression and conduct disorder and behavioral problems are available. A systematic review and meta-analysis of the association between childhood maltreatment and long-term outcomes (Norman et al., 2012) suggested that children who experience physical abuse were 1.5 times more likely to develop depressive disorders, and 2 to 6 times more likely to develop conduct disorder and behavioral problems (depending on the detection sample; in non-representative and population-based samples respectively). While it is likely that the effect sizes observed in this study may be inflated by the relatively small sample size, results suggest that air pollution represents an environmental risk factor that warrants further investigation with respect to depression and conduct disorder in young adults.

We found an increased likelihood of depression and conduct disorder at 18 in those exposed to higher estimated pollution levels (PM_2.5_ and NO_2_) at age 12, but no significant association with concurrent depression or conduct disorder symptoms. These results suggest that earlier exposure to NO_2_ and PM_2.5_ may each represent important risk factors for the development of these disorders at later ages. This may reflect the cumulative effect of chronic exposure to air pollution by age 18, or it could be that air pollution exposure takes time to impact on the processes underlying such behavioral problems. Many aspects of brain maturation and development continue during late childhood and adolescence (Rice and Barone, 2000), particularly emotion regulation (Steinberg, 2005), and therefore environmental exposures during this time have the potential to influence this process (e.g. via inflammatory pathways). Increased levels of neuroinflammation and markers of neurodegeneration have been reported in the brains of children and young adults exposed to high levels of air pollution (Calderon-Garciduenas et al., 2008), and thus it is conceivable that early or cumulative exposure to air pollution may exert long-term physiological and behavioral effects. It is also possible that exposure to air pollution during late childhood and adolescence is indicative of other, unmeasured factors with potentially important effects on depression and conduct disorder.

### 4.1 Strengths and limitations

In this study, we combined high-resolution air pollution concentration exposure estimates with densely phenotyped cohort data. Mental health was measured longitudinally and assessed using validated measures at an individual level, considering symptom-level and diagnostic outcomes. In addition, associations were tested for robustness to potentially important confounders such as sex, neighborhood- and family-level SES, smoking, family psychiatric history, childhood victimization, and age-12 mental health problems (in the age-18 analyses). However, there are several caveats that should be considered when interpreting the results. Firstly, the analyses in this study were restricted to E-Risk participants who lived in Greater London due to the availability of high-resolution pollution data in this area. Consequently, the sample size was relatively small, limiting the statistical power to detect associations between air pollution exposure and complex phenotypic outcomes. Additionally, while exposure to pollution varies within London, it was not possible to compare across other urban and rural environments. In this sample, annualized concentrations were within the legal limit for PM_2.5_ (by EU legislature, but not according to the WHO guidelines), but were exceeded for NO_2_ in over 30% of participants. It should be taken into account that research suggests the risk of adverse outcomes is significantly elevated even at levels below these legal limits (Beelen et al., 2013). Secondly, pollution exposure estimates in this study were based on the children's residential addresses. A more accurate estimation of pollution exposure would be achieved by including other locations where the children spend a substantial proportion of their time, such as their school, but this information was not available. Thirdly, the sample comprised twins, and so the findings may not generalize to singletons. However, the prevalence of mental health and behavioral problems has been shown to be the same for twins and singletons (Gjone and Nøvik, 1995). Fourthly, it is possible that genetic factors may influence an individual's biological response to air pollution exposure and subsequent development of mental health problems. Although we controlled for family psychiatric history in our analyses, this is only a proxy for genetic factors and future studies should control for more direct measures of genetic liability (e.g. polygenic risk scores). Finally, while we controlled for a number of important potential confounders, it is possible that further factors may confound the relationship between pollution exposure and mental health problems, such as noise pollution, exposure to indoor pollutants (e.g. parental smoking in the home, log fires), exacerbation by physical health problems, availability of green space, population density, or other factors associated with or that vary within urban environments, which we were not able to control for in the current study. Ideally, future studies should utilize discordant twin designs, where twins within a pair have been exposed to different levels of air pollution and the associations are then estimated in relation to later mental health problems between these differentially exposed twins, to maximize control for unmeasured shared environmental and genetic factors.

### 4.2 Conclusions

We have utilized high-resolution, individual-level data to explore for the first time the association between exposure to two main air pollutants and facets of childhood and adolescent mental health both cross-sectionally and longitudinally in a London-based subsample of a UK birth cohort. This pilot study highlights the utility of incorporating high-resolution modelling estimates of individuals’ environmental exposures with prospectively-collected phenotypic data from a longitudinal cohort and may serve as an exemplar for future investigations in other cohort studies. We found no evidence of associations between air pollution exposure in childhood and concurrent mental health problems at age 12. However, associations were apparent for subsequent development of symptoms and clinically diagnosable depression and conduct disorder at age 18. These findings warrant further investigation in larger, population-based cohort studies across urban and rural settings, with comprehensive assessments of exposures and outcomes at different developmental stages.

## Funding

The E-Risk study is funded by the UK Medical Research Council (G1002190). Additional support was provided by the National Institute of Child Health and Human Development (HD077482); the Jacobs Foundation; the Department of Health via the National Institute for Health Research Comprehensive Biomedical Research Centre award to Guy's and St. Thomas’ National Health Service (NHS) Foundation Trust in partnership with King's College London and King's College Hospital NHS Foundation Trust; Google Streetview; British Academy (SQ140024); a NERC-MRC-CSO grant (NE/P010687/1); and an MQ Fellows award to H.L.F. (MQ14F40). C.LO. is a Jacobs Foundation Advanced Research Fellow. L.A. is the Mental Health Leadership Fellow for the UK Economic and Social Research Council. Disclaimer: The views expressed are those of the authors and not necessarily those of the National Health Service, the National Institute for Health Research, or the Department of Health.

## Conflicts of interest

None

## Appendix Supplementary materials

Image, application 1
