# Supplementary material for: Exploration of NO2 and PM2.5 air pollution and mental health problems using high-resolution data in London-based children from a UK longitudinal cohort study
Source: Psychiatry Res. 2019 Feb;272:8–17. doi: 10.1016/j.psychres.2018.12.050 (PMC6401205; doi:10.1016/j.psychres.2018.12.050)
Supplement: Supplementary file 1 [file mmc1.docx]

**Supplementary Material**

**Table S1.** Comparison of demographic characteristics between the London-based subsample and the rest of the Environmental Risk (E-Risk) sample.

| **Characteristic** | | **Study members living in London**  **(*N*=284)**  ***n* (%) or *M* (SD)** | **Study members living outside of London**  **(*N*= 1948)**  ***n* (%) or *M* (SD)** | **Statistical test results** |
| --- | --- | --- | --- | --- |
| Male | | 152 (53.5) | 940 (48.3) | χ^2^(1, 2232)=2.75, *p*=0.097 |
| Monozygotic twins | | 144 (50.7) | 1102 (56.6) | χ^2^(1, 2232)=3.46, *p*=0.063 |
| Ethnicity | White | 170 (59.9) | 1848 (94.9) | Fisher’s exact test *p*<0.001 |
|  | Asian | 34 (12.0) | 56 (2.9) |  |
|  | Black | 36 (12.7) | 6 (0.3) |  |
|  | Mixed race | 4 (1.4) | 4 (0.2) |  |
|  | Other | 40 (14.1) | 34 (1.75) |  |
| Family SES | High | 116 (40.9) | 636 (32.7) | χ^2^(2, 2232)=9.10, *p*=0.011 |
|  | Medium | 92 (32.4) | 646 (33.2) |  |
|  | Low | 76 (26.8) | 666 (34.2) |  |
| Neighborhood SES | Wealthy Achievers | 50 (17.7) | 498 (26.8) | χ^2^(4, 2138)=219.70, *p*<0.001 |
|  | Urban Prosperity | 66 (23.4) | 48 (2.6) |  |
|  | Comfortably Off | 56 (19.9) | 576 (31.0) |  |
|  | Moderate Means | 30 (10.6) | 256 (13.8) |  |
|  | Hard Pressed | 80 (28.4) | 478 (25.8) |  |
| Ever a daily smoker | | 50 (20.0) | 491 (27.1) | χ^2^(1, 2065)=5.65, *p*=0.017 |
| Proportion of family members with a history of psychiatric disorder (0-1) | | 0.34 (0.29) | 0.38 (0.27) | *t*(2136)=1.98, *p*=0.048 |
| Exposed to severe victimization in childhood | | 73 (25.7) | 518 (26.6) | χ^2^(1, 2232)=0.10, *p*=0.752 |

*M*, mean. SD, standard deviation. SES, socioeconomic status.

**Table S2.** Phenotypic outcomes at ages 12 and 18: means and distributions.

| **Phenotypic Observation** | | **Age** | **Mean (SD)** | **Range** | **Percent**  ***n* (%)** |
| --- | --- | --- | --- | --- | --- |
| Anxiety | Symptom scale | 12 | 7.88 (2.90) | 1-18 | - |
|  |  | 18 | 0.82 (1.69) | 0-6 |  |
|  | Diagnosis | 18 |  | | 16 (6.4) |
| Depression | Symptom scale | 12 | 2.95 (5.09) | 0-30 | - |
|  |  | 18 | 1.68 (2.82) | 0-9 |  |
|  | Diagnosis | 18 |  | | 47 (18.8) |
| Conduct Disorder | Symptom scale | 12 | 1.93 (2.00) | 0-8 | - |
|  |  | 18 | 2.22 (2.39) | 0-11 |  |
|  | Diagnosis | 18 |  | | 39 (15.6) |
| ADHD | Symptom scale | 12 | 11.49 (10.18) | 0-42 | - |
|  |  | 18 | 0.53 (1.20) | 0-6 |  |
|  | Diagnosis | 18 |  | | 17 (6.8) |

ADHD, Attention Deficit Hyperactivity Disorder. SD, standard deviation.

**Table S3.** Air pollution distribution by neighborhood-level socioeconomic status (classified using ACORN).

| **Pollutant** | **Neighborhood classification** | **Mean (SD)** | **Statistical test result** |
| --- | --- | --- | --- |
| PM_2.5_ | Wealthy achievers | 13.49 (0.35) | *F*(4,277)=32.06, *p*<0.001 |
|  | Urban prosperity | 14.48 (0.62) |  |
|  | Comfortably off | 13.81 (0.37) |  |
|  | Moderate means | 13.87 (0.41) |  |
|  | Hard-pressed | 14.43 (0.78) |  |
| NO_2_ | Wealthy achievers | 33.00 (3.16) | *F*(4,277)=31.38, *p*<0.001 |
|  | Urban prosperity | 41.20 (5.07) |  |
|  | Comfortably off | 35.98 (3.60) |  |
|  | Moderate means | 35.81 (3.28) |  |
|  | Hard-pressed | 40.30 (5.96) |  |

ACORN, A Classification of Residential Neighborhoods; SD, standard deviation.
